# Case Report: Dental treatment for an oboist: Post-trauma prosthetic rehabilitation and evaluation of musical performance

**DOI:** 10.3389/fpsyg.2022.1022205

**Published:** 2023-02-01

**Authors:** Mariko Hattori, Sebastian B. M. Patzelt, Michiichiro Itoh, Yuka I. Sumita, Noriyuki Wakabayashi

**Affiliations:** ^1^Advanced Prosthodontics, Tokyo Medical and Dental University, Tokyo, Japan; ^2^Medical Center, Center for Dental Medicine, Department of Prosthetic Dentistry, Faculty of Medicine, University of Freiburg, Freiburg, Germany; ^3^Private Dental Clinic – Dres. Patzelt, Zimmern ob Rottweil, Germany; ^4^Bancho Oral Surgery and Scanning, Tokyo, Japan

**Keywords:** dentistry, prosthodontics, trauma, implant, musician, oboe

## Abstract

**Introduction:**

The condition of teeth and function of the oral organs are important when playing wind or brass instruments. Although there are some reports on dental treatment for musicians, few studies have investigated their acoustic performance following treatment. This report describes the prosthodontic rehabilitation provided for an oboist who had lost a tooth as a result of trauma and includes an evaluation of her subsequent musical performance using acoustic analyzes.

**Case description:**

The patient was a 63-year-old professional oboe player who fractured the upper and lower alveolar bone and avulsed the upper right central incisor during a fall due to epileptic seizure. While the alveolar fracture was healing, she sought maxillofacial rehabilitation for the missing tooth to maintain her ability to play the oboe. Her rehabilitation consisted of a provisional removable prosthesis with an acrylic base and clasps followed by a fixed implant prosthesis. A recording of her musical performance was objectively analyzed at each stage of treatment. Rhythm analysis confirmed the stability of notes played rapidly. Her performance dynamics were analyzed by psychoacoustic measurements. Her satisfaction with the prosthesis was assessed by a self-reported questionnaire. The results of the acoustic evaluation helped to adjust the provisional prosthesis so that it was suitable for playing the oboe and the final prosthesis was designed accordingly.

**Conclusion:**

Prosthetic dental treatment for this patient included both subjective and objective evaluations that helped to ensure that she could continue playing the oboe at her previous performance level.

## 1. Introduction

Teeth have an important role in playing brass, woodwind, and other instruments (Woldendorp et al., 2016). According to recent reviews, musicians can experience occupational orofacial problems caused by excessive practice and stress (Rodríguez-Lozano et al., 2011). Dentists may address these issues (Yeo et al., 2002). Furthermore, malocclusion can affect wind instrumentalists’ performance and embouchure comfort (van der Weijden et al., 2018). A reported case demonstrated that a professional horn player’s playing was improved by dental correction (van der Weijden et al., 2019). These reports involved younger musicians; however, older musicians may also benefit from dental treatment of age-related orofacial issues (e.g., missing teeth) interfering with their performance ability.

In an experimental study of musical performance, objective acoustic analyzes could reveal changes in the oral cavity (Hattori et al., 2014). A case of prosthetic treatment in an older clarinetist with missing teeth demonstrated that objective evaluation of musical performance can aid prosthetic adjustments to facilitate playing the clarinet (Hattori et al., 2015). Another report described objective evaluations of woodwind performance of a child who needed prosthetic rehabilitation after a tumor resection (Elbashti et al., 2018).

This report describes a prosthetic rehabilitation in an older professional oboist. She was an experienced music teacher and lost a front tooth due to trauma. The prosthodontist objectively assessed her musical performance using acoustic analyzes which guided the prosthetic treatment.

## 2. Case description

A 63-year-old woman visited our clinic in 2012 complaining about difficulties in playing the oboe following an accident. She had been an oboe, recorder, and piano teacher for 40 years. Apart from otitis media and hypertension, she had had no known health issues until a week earlier, when she had fallen at home due to an epileptic seizure. She fractured the upper and lower alveolar bone, avulsed and lost the upper right central incisor. Immediately afterward, an oral surgeon reduced the fractures, splinted the teeth using dental adhesive resin cement, and sutured the ruptured gingiva and lip. Seven days later, she was referred to the Department of Maxillofacial Prosthetics, Graduate School, Tokyo Medical and Dental University (TMDU), Tokyo, Japan for dental treatment.

**Figure 1:**
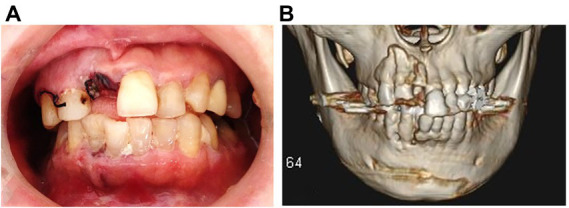
**(A)** Intraoral view at the patient’s first visit (5.7.2012). **(B)** Three-dimensional computed tomography scan obtained before treatment (5.7.2012).

She could walk and speak normally. She felt a little pain around the trauma site but had hypoesthesia on the right lower lip and the surrounding area. The upper right central incisor was missing (Figure 1A) and there was a lateral luxation of the upper right lateral incisor and canine and the lower right incisors and canine. However, these teeth were well splinted with limited mobility. The lower right central incisor was not sensitive to a cold stimulus while all other remaining teeth were except for the upper left lateral incisor with root canal filling. Radiographs showed alveolar fractures around the apices of the upper and lower right incisors and the upper right canine (Figure 1B). No temporomandibular joint disorder was found.

Options for definitive prosthetic rehabilitation were explained to the patient including removable prosthesis, fixed partial denture, or a fixed implant prosthesis. She chose a fixed implant prosthesis, and early insertion of the implant fixture was planned.

**Figure 2:**
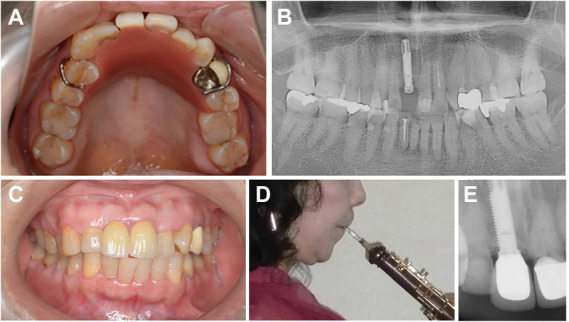
**(A)** Occlusal view with the removable prosthesis placed (19.7.2012). **(B)** Panoramic radiograph with the dental implant inserted (19.7.2012). **(C)** Intraoral view with the final prosthesis in place (7.3.2013). **(D)** Lateral view of the patient with the oboe in place (7.3.2013). **(E)** Dental radiograph after 8 years observation (2.9.2021).

Immediately after taking an impression and jaw registration, a removable prosthesis consisting of an artificial tooth and acrylic base was inserted and adjusted. The patient was advised to start with easy practice and playing. The prosthesis had an artificial tooth, acrylic base, and two wire clasps on both first premolars. When the prosthesis was tried, the acrylic base on the palate was reduced according to performance and patient preference (Figure 2A).

Two weeks later, a titanium dental implant (Brånemark System Mk III RP 3.75 × 18 mm, Nobel Biocare, Gothenberg, Sweden) was placed in the position of the missing incisor (Figure 2B). The implant achieved bicortical anchorage with adequate primary stability. Four months later, an acrylic provisional superstructure was tried and adjusted in the clinic while the patient played the oboe. The incisal curve was fitted with a carbide bar and polished. The provisional superstructure was then screwed in. A zirconia abutment and final superstructure made of layered zirconia with a ceramic veneer was then created in the shape of the provisional restoration (Figure 2C). The patient again played the instrument in the clinic to confirm the contour before the final prosthesis was cemented (Figure 2D).

A self-reported questionnaire was used to assess the patient’s sensation of the stability and comfort of the removable prosthesis, fit of the oboe reed, blowing comfort, sound quality, and overall satisfaction. Each item was assessed for the removable prosthesis before and after adjustment and the provisional and final superstructures of the implant prosthesis. For comparison, the patient retrospectively assessed each item before the injury.

**Figure 3:**
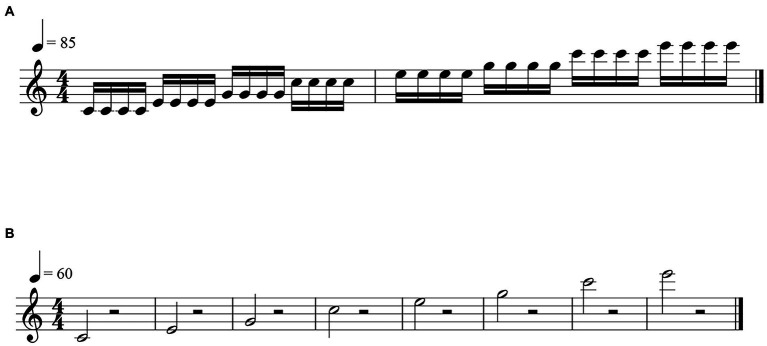
**(A)** Test tone used in the rhythm analysis. **(B)** Test tone used in the dynamic analysis.

Musical performance was assessed using an acoustic analysis system when the provisional removable prosthesis was tried in/adjusted and when the final prosthesis was seated. Her tonguing control was assessed with rhythm analyzes (Hattori et al., 2015) by recording the notes shown in mezzo forte at 85 beats per minute (Figure 3A). A microphone (SM58, Shure, Tokyo, Japan) was placed 10 cm from the oboe’s bell and the performance was recorded with a speech analysis system (Computerized Speech Lab 4,400, Kay Pentax, Lincoln park, NJ, United States). For each note, the moment of tonguing was recorded using speech analysis software (Computerized Speech Lab Main Program; Kay Pentax), and the interval to the next tonguing was used to examine the length of each note. Although rhythm needs to be flexible for expression, precise timing is important to play (Hattori et al., 2015). The coefficient of variation (CV) for note length was calculated to determine the stability of the rhythm. The recording was randomly played back and listened by a music expert. Narrative evaluation of the performance was recorded.

As precise control of dynamics is also important for expressive performance, performance in three different dynamics was evaluated using psychoacoustic analyzes (Hattori et al., 2014, 2015). A microphone (LA5120, Ono Sokki, Kanagawa, Japan) was placed 20 cm from the oboe’s bell, and the notes (Figure 3B) were played pianissimo, mezzo forte, and fortissimo at 60 beats per minute while wearing the final prosthesis. The patient’s performance was recorded using recording software (Osreco, Ono Sokki) and analyzed using a psychoacoustic system (Oscope 2, Ono Sokki). The loudness (Zwicker et al., 1957) and sharpness (Bismark, 1974) of each note were used to evaluate performance.

## 3. Results

**Figure 4:**
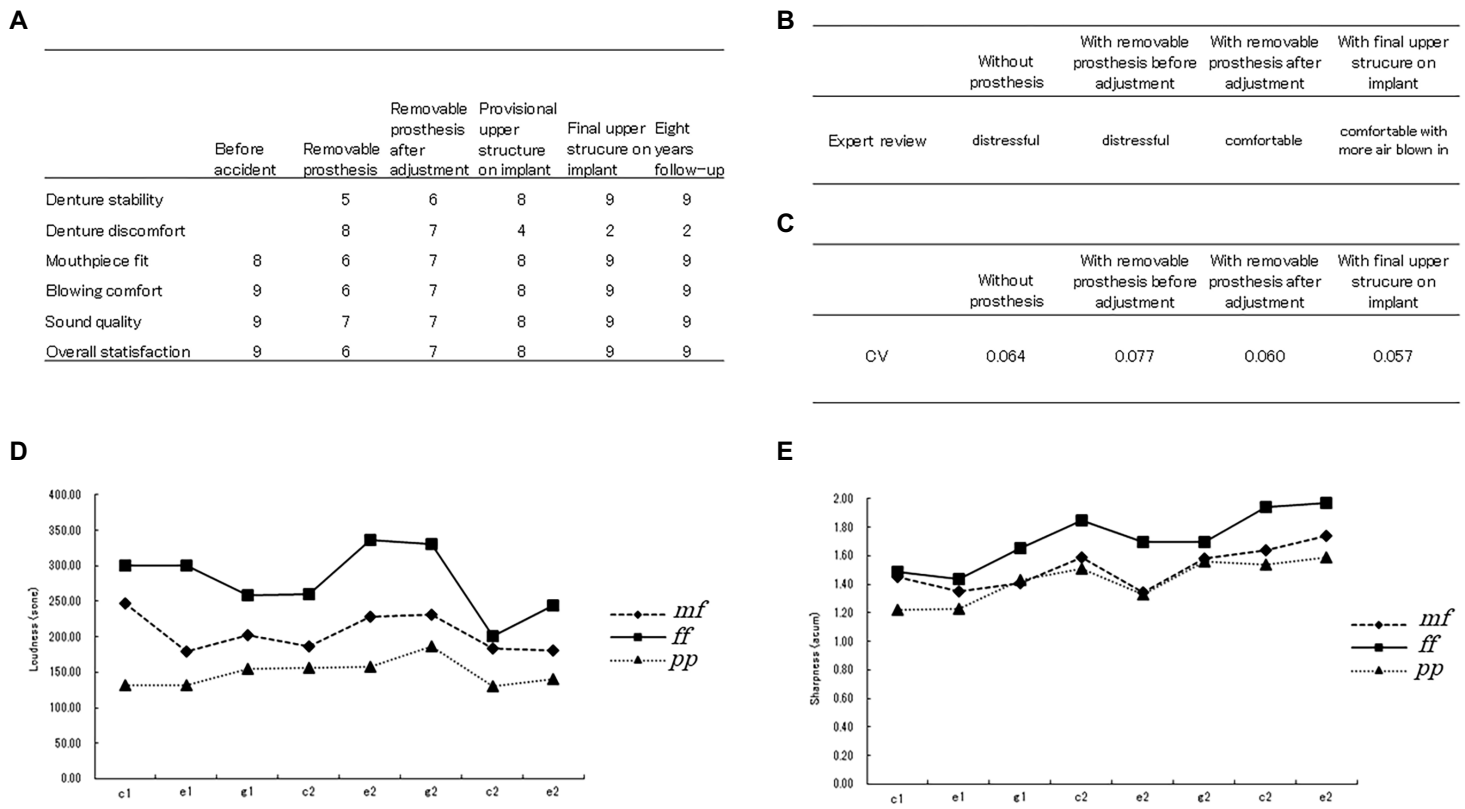
Results of this study. **(A)** Patient’s self-reported rating of satisfaction with her prosthetic rehabilitation and musical performance. **(B)** Results of the expert review. **(C)** Coefficient of variation when rhythm analysis was performed at each step of rehabilitation. **(D)** Results of loudness analysis of notes played by the oboe in three different dynamics. mf, mezzo forte (moderately loud); ff, fortissimo (very loud); pp., pianissimo (very quiet). **(E)** Result of sharpness analysis of notes played by the oboe in three different dynamics. mf, mezzo forte; ff, fortissimo; pp., pianissimo.

The hypoesthesia lessened in the first 2 months and had disappeared entirely by 1 year after the accident. The removable prosthesis and final implant-supported prosthesis fit well and were stable. The patient was able to eat, swallow, and speak well. There was no pain or irritation in the surrounding tissue and the esthetic outcome was excellent. The patient felt discomfort with the removable prosthesis but this diminished when seating the implant prosthesis. The patient rated the final prosthesis suitable for playing the oboe (Figure 4A).

The expert review showed that the recording sounded comfortable and with more air blown in when final prosthesis was worn (Figure 4B).

The CV for note length while trying to play a steady rhythm was larger with the removable prosthesis than without it but decreased when the prosthesis was adjusted. The CV was the smallest with the final prosthesis (Figure 4C).

Figures 4D,E show psychoacoustic evaluation results for loudness and sharpness, respectively, and confirm that the three dynamics were well differentiated.

During 8 years of follow-up after delivery of the final prosthesis, there were no occlusal changes or any bone resorption around the implant (Figure 2E). The patient did not notice any change when playing the oboe (Figure 4A) and continues to practice, teach, and perform music with the final prosthesis.

## 4. Discussion

The best option to save an avulsed tooth is an immediate reimplantation (Andersson et al., 2017). However, this was not possible in the present patient. An appropriate emergency treatment was provided. Early insertion of an implant fixture was selected because of unsuitable abutment teeth for a fixed denture and because of the need to prevent excessive bone resorption and deformation of the alveolar ridge. A removable prosthesis with an acrylic base, an artificial tooth and clasps was used for a provisional restoration. A cantilever restoration was considered as not useful due to the condition of the neighboring teeth and the risk of extensive force on the restoration during playing the oboe. A partial denture with a rest and casted clasps was not used either, although a stable design with correct retention and support is usually recommended for prosthetic rehabilitation. The clasps were on the premolars, which were intact. The front teeth were avoided considering embouchure or tonguing. The patient made an early return to music even with the removable prosthesis. The final implant-supported prosthesis restored the defect well allowing recovery of mastication, speech, and appearance. Furthermore, it was stable and strong enough to withstand the force of playing the oboe. However, the amount and angulation of loads differ between playing music and mastication, and it is not known whether unwanted forces act on the implant while playing. The observation of the implant and the circumferential bone over a period of 8 years showed no remarkable change.

Our patient perceived differences in stability among the prostheses, possibly because the displacement of the removable prosthesis caused by the pressure of the mouthpiece. Discomfort of the removable prosthesis could be explained by the acrylic base on the lingual side where tonguing is performed. In her case, the tooth loss was trauma-related and there was no bone defect, which is why the acrylic base interfered with the performance. Her overall satisfaction was primarily based on the mouthpiece fit and blowing comfort. The acoustic analyzes showed that the patient could play at a relatively high level even without a prosthesis or with the adjustable prosthesis, but more effort was required to achieve a given level of performance resulting in less comfortable playing.

Her tonguing control was assessed by rhythm analyzes. Her control was worst with the removable prosthesis before adjustment. Possibly, it was due to the acrylic base in the maxilla made tonguing difficult to control owing to its thickness and loss of sensory feedback from the oral mucosa. Her tonguing became more stable after a base adjustment indicating that the loss of the central incisor affected her playing. The patient showed the best control of rhythm when the final implant-supported prosthesis was seated.

Psychoacoustic analyzes (Zwicker et al., 1957) showed that the notes played in three dynamics were well differentiated in terms of loudness and sharpness. Loudness is the psychoacoustic counterpart to sound pressure level, and sharpness is the balance of high and low frequencies perceived by the human ear (Zwicker et al., 1957; Bismark, 1974). Human hearing has specific characteristics including sensitivity to certain frequencies and a masking effect; thus perception of music is affected by both, physical quantities and our hearing mechanisms.

In the present patient, the evaluation was performed soon after the prosthesis was tried or inserted. A chronological evaluation over longer periods should be investigated in the future. Notably, the patient returned to music even under imperfect conditions. This suggests that patients should be encouraged to return playing there instrument as soon as possible even under imperfect conditions. However, a prompt return to music does not necessarily correspond to full satisfaction. Dentists should always aim for ongoing rehabilitation including adjusting the prosthesis or seeking a further prosthetic treatment, but irreversible treatments should be performed with caution and informed consent.

## 5. Conclusion

Subjective and objective evaluations of a musician’s ability to play their instrument in prosthetic rehabilitation can help dentists to assess performance levels, the treatment satisfaction of the patient and optimize the treatment.

## Data availability statement

The original contributions presented in the study are included in the article/supplementary material, further inquiries can be directed to the corresponding author.

## Ethics statement

The studies involving human participants were reviewed and approved by Ethics Committee of the Faculty of Dentistry, Tokyo Medical and Dental University (approval no. 579 and no. D2016-088). The patient provided her written informed consent to participate in this study. Written informed consent was obtained from the individual for the publication of any potentially identifiable images or data included in this article.

## Author contributions

MH and YS: conceptualization. MH and MI: treatment. MH: psychoacoustic analysis. SP: validation. MH, SP, MI, and YS: investigation. MH: data curation and writing – original draft preparation. YS: writing – review and editing. NW: supervision and project administration. All authors contributed to the article and approved the submitted version.

## Conflict of interest

The authors declare that the research was conducted in the absence of any commercial or financial relationships that could be construed as a potential conflict of interest.

The authors declare that this study received funding from Yamaha Corporation. The funder had the following involvement in the study: study design and decision to publish.

## Publisher’s note

All claims expressed in this article are solely those of the authors and do not necessarily represent those of their affiliated organizations, or those of the publisher, the editors and the reviewers. Any product that may be evaluated in this article, or claim that may be made by its manufacturer, is not guaranteed or endorsed by the publisher.
